# Effectiveness of Sulfadoxine–Pyrimethamine for Intermittent Preventive Treatment of Malaria and Adverse Birth Outcomes in Pregnant Women

**DOI:** 10.3390/pathogens9030207

**Published:** 2020-03-11

**Authors:** Eulambius M. Mlugu, Omary Minzi, Muhammad Asghar, Anna Färnert, Appolinary A.R. Kamuhabwa, Eleni Aklillu

**Affiliations:** 1 Department of Pharmaceutics, School of Pharmacy, Muhimbili University of Health and Allied Sciences, Dar es Salaam 0702172, Tanzania; mlugusonlove@gmail.com; 2 Division of Clinical Pharmacology, Department of Laboratory Medicine, Karolinska Institutet at Karolinska University Hospital, 141 86 Stockholm, Sweden; 3 Department of Clinical Pharmacy and Pharmacology, School of Pharmacy, Muhimbili University of Health and Allied Sciences, Dar es Salaam 0702172, Tanzania; minziobejayesu@gmail.com (O.M.);; 4 Division of Infectious Diseases, Department of Medicine Solna, Karolinska Institutet, 171 76 Stockholm, Sweden; asghar.muhammad@ki.se (M.A.); anna.farnert@ki.se (A.F.); 5 Department of Infectious Diseases, Karolinska University Hospital, 171 76 Stockholm, Sweden

**Keywords:** malaria in pregnancy, IPTp-SP, adverse birth outcomes, low birth weight, anemia, Tanzania

## Abstract

Effectiveness of intermittent preventive treatment in pregnancy with sulfadoxine–pyrimethamine (IPTp-SP) for prevention of malaria and adverse birth outcomes can be compromised by parasites-resistance to sulfadoxine–pyrimethamine. This study prospectively evaluated the effectiveness of IPTp-SP in Southeast Tanzania. From January 2017 to May 2019, HIV-negative and malaria-negative (mRDT) pregnant women attending their first antenatal-care visit in the second or third trimester (n = 500) were enrolled to receive monthly IPTp-SP and followed the protocol till delivery. The primary outcome was the prevalence of histopathological placental malaria. Secondary outcomes were anemia, malaria parasites detected during pregnancy and at delivery, adverse birth outcomes (low-birth-weight [LBW], premature birth, fetal anemia, still birth, and spontaneous abortion). Rates of histopathological placental malaria, any parasitemia at delivery (placental, cord or maternal), and any adverse birth outcome were 9.4%, 20.9%, and 26.5%, respectively. Rates of symptomatic malaria and parasitemia during pregnancy were 2.8% and 16%, respectively. Histopathological placental malaria significantly increased the odds of any adverse birth outcomes, particularly LBW. IPTp-SP with more than or equal to three doses significantly improved birth weight and reduced the risk of LBW by 56% compared to <3 SP doses (*p* = 0.009). IPTp-SP with more than or equal to three doses is still effective in improving birth weight. However, the detection of histopathological placental-malaria in one-tenth and parasitemia in one-fifth of pregnant women reflects the need to optimize the prevention of malaria during pregnancy.

## 1. Introduction

Malaria during pregnancy is a public health problem causing maternal anemia and placental insufficiency leading to preterm delivery, stillbirth and low birth weight (LBW) [1,2,3]. Each year, more than 30 million of all women who become pregnant in Sub-Saharan Africa are at risk of malaria [4]. Pregnant women are more susceptible to malaria and its consequences than non-pregnant women due to pregnancy-related decreased immunity as well as placental specific variants adhesion of *P. falciparum* erythrocyte membrane protein 1 (PfEMP1) to chondroitin sulfate A (CSA) in the placenta [5,6].

Due to the high risk and burden of malaria among pregnant women in endemic areas, the World Health Organization (WHO) has developed prevention strategies to improve pregnancy outcomes. The strategic package includes sleeping under insecticide-treated nets, indoor residual spraying, effective case management, and intermittent preventive treatment in pregnancy with sulfadoxine–pyrimethamine (IPTp-SP) starting early in the second trimester [7]. Monthly IPTp-SP is now recommended throughout pregnancy in endemic countries [8].

The WHO policy on IPTp-SP considers three and more doses of SP given monthly for intermittent preventive treatment in pregnancy (IPTp-SP 3+) to be optimal, and less than three doses as sub-optimal [9]. The IPTp-SP 3+ policy has been slowly but fully adopted and implemented in sub-Saharan Africa, including Tanzania [10]. Studies indicate that IPTp-SP 3+ offers more malaria prevention over sub-optimal doses. For instance, it has been shown to improve birth weight and reduce the risk of LBW [11,12]. However, in most sub-Saharan African countries, pregnant women begin first antenatal care (ANC) visit late [13,14]. Therefore, most women do not benefit fully from the services provided, including receiving optimum IPTp-SP for the prevention of malaria and adverse birth outcomes. Other factors such as poor attendance to ANC contribute to limited access to optimal IPTp-SP [15].

In Tanzania, only a few cross-sectional studies have investigated the effectiveness of IPTp-SP previously [16,17,18]. The effectiveness of IPTp-SP in sub-Saharan Africa is currently threatened by increasing *P. falciparum* resistance to SP. Resistance to SP is conferred by mutations in *P. falciparum dihydrofolate reductase (Pfdhfr)* and *dihydropteroate synthetase (Pfdhps)* genes [19,20]. The emergence of new additional mutations in these genes resulting in highly resistant parasites further increases concerns for the effectiveness of IPTp-SP [21,22]. Therefore, there is a need to continuously monitor the effectiveness of this useful intervention in the light of increasing *P. falciparum* resistance to SP. This is especially important due to the unavailability of suitable alternative drugs for intermittent preventive treatment in pregnancy (IPTp) at the moment. Therefore, we conducted a prospective observational study to evaluate the effectiveness of IPTp-SP given as directly observed therapy (DOT) and prospectively monitored the treatment outcomes throughout pregnancy and at delivery in an area of moderate malaria transmission in Southeast Tanzania.

## 2. Results

**Figure 1:**
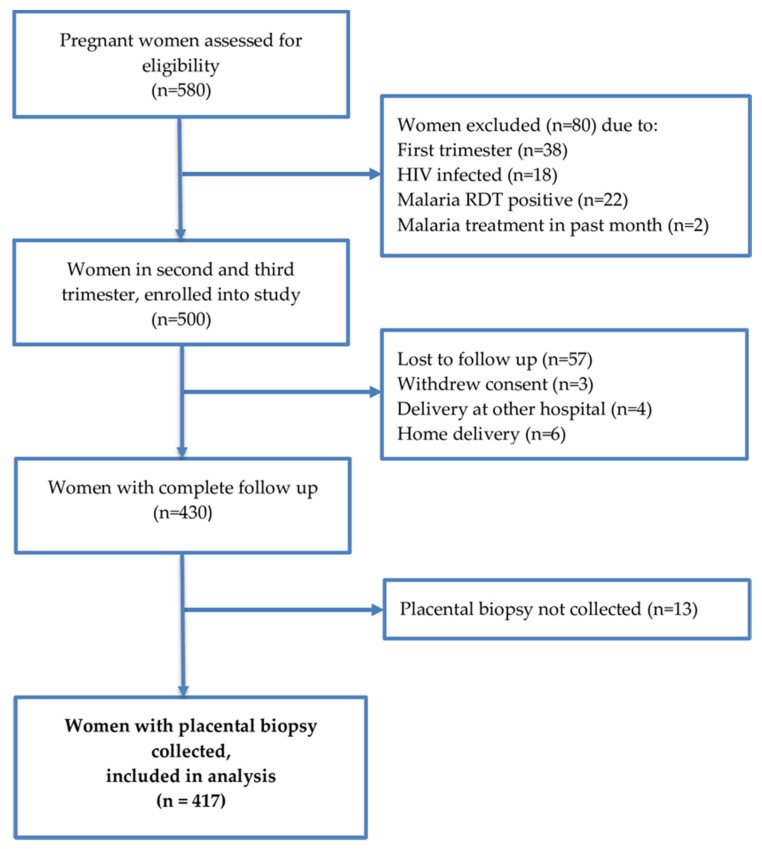
Study flow chart.

From January 2017 to May 2019, 500 pregnant women were recruited at their first antenatal care (ANC) visit and given monthly IPTp-SP (Figure 1). Enrolled participants were mainly women aged 20–34 years (Table 1). A total of 417 (83.4%) malaria-free women at enrolment were followed throughout pregnancy and had placental biopsies collected at delivery (Figure 1). The women had a median of three (min: one; max: five) ANC visits at which screening of malaria parasites (by microscopy, mRDT and real-time PCR) and determination of hemoglobin concentration were done followed by administration of SP under DOT. About two-thirds (61.4%, 256/417) of the study participants received optimal IPTp-SP doses (more than or equal to three doses), and 38.6% (161/417) received sub-optimal IPTp-SP doses (less than three doses). The newborn mean birth weight with one standard deviation was 2.9 (0.4) kg, and 10.9% (46/423) infants had LBW. Table 1 summarizes the characteristics of study participants at enrollment and during follow up.

### 2.1. Detection of Malaria Parasites at Delivery

**Figure 2:**
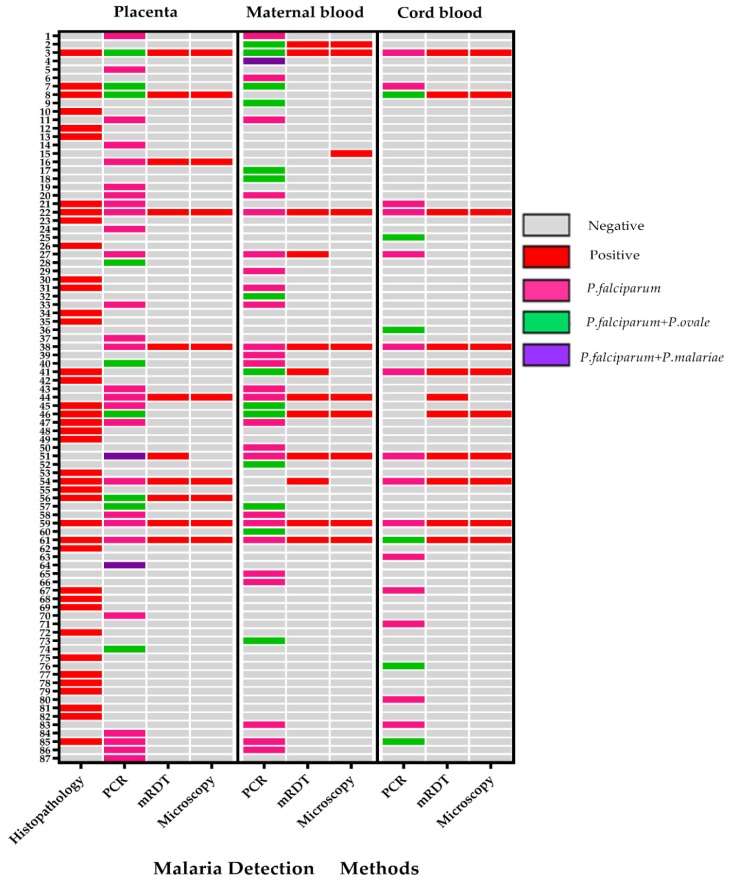
Heat map showing detection of any malaria (n = 87) at delivery in placental tissue; placental blood; maternal peripheral blood; and cord blood using microscopy, mRDT, and real-time PCR. Different malaria species detected by real-time PCR are also shown.

Screening for malaria parasites was done at delivery from placental tissue; placental blood; maternal venous blood; and cord blood using microscopy, mRDT, and real-time PCR (Table 2). Figure 2 summarizes all cases of malaria parasites detected at delivery using various methods. There was a strong correlation between mRDT and microscopy in the detection of malaria parasites (kappa = 0.95). Compared to real-time-PCR, the sensitivity and specificity of mRDT were 32.2% and 94.1%, and microscopy were 25.4% and 99.5%, respectively. In the placenta, the histopathological detection of malaria by microscopy had a sensitivity and specificity of 34.2% and 93%, compared to PCR of placental blood.

The overall prevalence of histopathological confirmed placental malaria (parasites and/or pigments) was 9.4% (39/417; [95% CI = 6.7 to 12.6]). Out of the 39 histopathological malaria cases, 29 (74%) participants had active infection as indicated by the presence of malaria parasites in the placental tissue; and 10 (26%) had only hemozoin pigments indicating past infection. There was no case with both parasites and pigments. Analysis of placental blood by mRDT, microscopy, and PCR detected parasites in 2.6% (11/417), 2.4% (10/417), and 9.1% (38/417) women, respectively. The overall prevalence of malaria parasites detected at delivery in either placental tissue (parasites and pigments), placental blood, maternal peripheral blood, or cord blood by mRDT, microscopy, and/or real-time PCR was 20.9% (87/417 [95% CI = 17.2 to 25.0]).

Using real-time PCR, *P. falciparum* mono-infection was the most predominant, accounting for 60% (91/153) of all the samples in 78 pregnant women during pregnancy and at delivery. Mixed species infection with *P. falciparum* and non-*P. falciparum* species accounted for overall 40% (62/153) in 55 pregnant women. In *P. falciparum* mixed infections, the combination with *P. ovale* was the most common accounting for 37% (56/153) in 49 pregnant women followed by *P. malariae* with 3% (6/153) in six pregnant women. Mono-infection alone with non-*falciparum* malaria was not detected in any sample.

#### 2.1.1. Factors Associated with Histopathological Placental Malaria

The association of histopathological placental malaria with various potential predictors was assessed. Compared to adolescent pregnant women (age < 20 years), young adult pregnant women aged 20–34 years had a 64% reduced risk of having placental malaria at delivery (OR. 0.36 [95% CI 0.16, 0.81], *p* = 0.014). This association remained significant in the multivariate analysis (Table 3). Being multigravida, having at least primary education, and receiving optimal SP doses (more than or equal to three doses) were not associated with significantly reduced odds of placental malaria. In addition, malaria during pregnancy and treatment with artemether-lumefantrine during pregnancy were not significantly associated with placental malaria at delivery (Table 3).

#### 2.1.2. Factors Associated with Detection of Malaria Parasites at Delivery

Various potential predictors of detection of parasites in any material and method of detection used at delivery were evaluated. Compared to adolescent pregnant women (age < 20 years), adult women (age ≥ 20 years) were not significantly associated with reduced odds of malaria parasites detection at delivery (OR 0.90 [95% CI 0.48, 1.60] *p* = 0.23). Primigravida and lack of formal education were not associated with significantly higher odds of malaria parasite detection at delivery compared to multigravida and having at least primary education (OR [95% CI]: 1.05 [0.62, 1.82] *p* = 0.85; and 1.47 [0.78, 2.86] *p* = 0.24) respectively. Moreover, detection of malaria during pregnancy and treatment with artemether-lumefantrine (stratified by sub-optimal SP doses [less than three doses] versus optimal SP doses [more than or equal to three doses]) was not associated with significantly reduced odds of any malaria at delivery (OR 0.47 [95% CI 0.14, 1.60] *p* = 0.23).

### 2.2. Malaria Parasites during Pregnancy after Initiation of IPTp-SP

Rates of malaria parasites detection during pregnancy identified by microscopy, mRDT and/or real-time PCR, is presented in Table 2. After initiation of IPTp-SP, 2.8% (14/500 [95% CI 1.5 to 4.7]) of women experienced a symptomatic episode of malaria (identified by mRDT and confirmed by microscopy) and received artemether-lumefantrine treatment. Asymptomatic infections were identified at regular scheduled ANC visits by mRDT and/or real-time PCR in 16% (80/500 [95% CI 12.9 to 19.5]) of women, including the whole period before delivery. Pregnant women who had ≥3 ANC visits had more malaria testing performed, and hence had also more cases of asymptomatic malaria detected compared to those who had lower (<3) visits (21% [54/256] versus 16.1% [26/161]).

#### Factors Associated with Malaria during Pregnancy after Initiation of IPTp-SP

**Figure 3:**
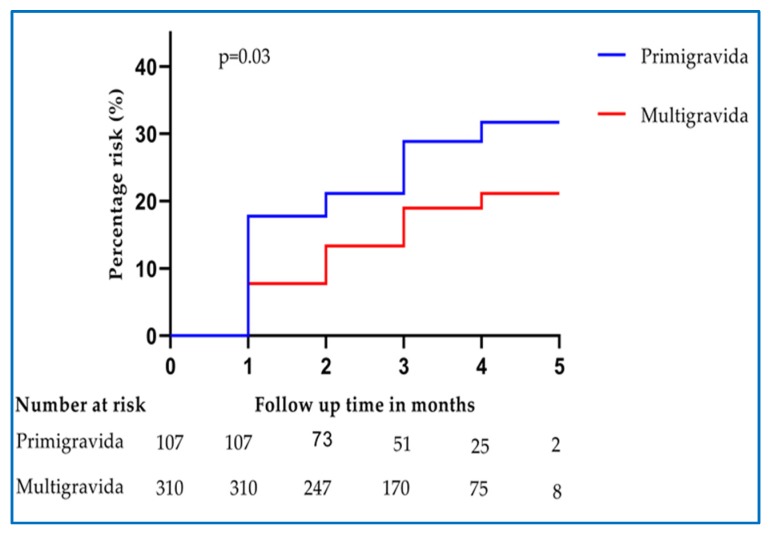
Hazard proportions for asymptomatic malaria positivity at ANC visits during pregnancy after the first dose of IPTp-SP stratified by gravidity.

Various potential predictors of asymptomatic malaria detected during pregnancy were assessed. In univariate Cox regression analysis, women who were pregnant for the first time (primigravida) were found to have a significantly higher (66%) risk of having malaria parasitemia during pregnancy compared to those who were pregnant for more than once (multigravida) (Table 3 & Figure 3). In multivariate analysis, the risk of having malaria parasitemia among primigravida has significantly increased to 70% compared to multigravida. At 1 month after initiation of IPTp-SP, the prevalence of parasitemia was significantly higher among primigravida 22 (20.6%) than in multigravida 30 (9.7%), *p* = 0.003. Age and education level did not significantly affect the risk of malaria parasites detected during pregnancy (Table 3).

### 2.3. Anemia during Pregnancy

The overall prevalence of anemia (Hb < 11 g/dL) during pregnancy was 73.8% (369/500). At enrollment, the prevalence of anemia was 57.2% (286/500). During follow up, 16.6% (83/500) of participants developed anemia. All cases of anemia during pregnancy were mild to moderate (Hb = 7–10.9 g/dL). At delivery, the prevalence of maternal anemia was 81% (337/417). Seven cases (1.7% [7/417]) of severe anemia were found at delivery. Two pregnant women with severe anemia at delivery had asymptomatic parasitemia. The majority of pregnant women who had anemia at enrollment (83% [237/286]) also had anemia at delivery (McNemar test *p* < 0.001).

#### Factors Associated with Anemia during Pregnancy

**Figure 4:**
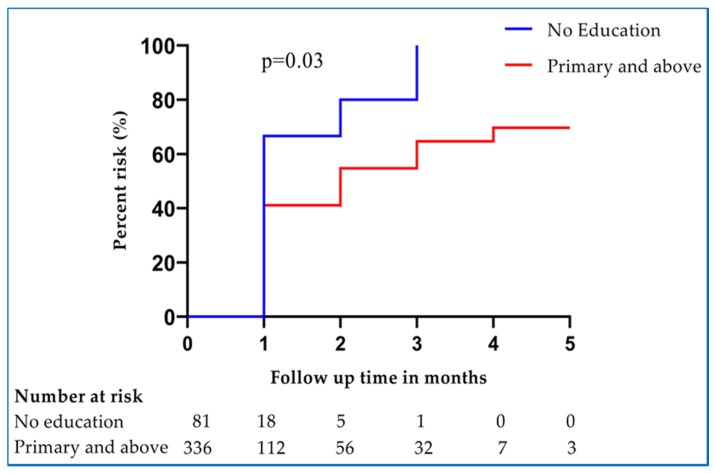
Hazard proportions for anemia during pregnancy after the first dose of IPTp-SP stratified by the level of education.

Various potential predictors of anemia during pregnancy were assessed. Women who were pregnant for the first time (primigravida) had no significant risk of having anemia compared to those women who were pregnant more than once (multigravida). Age was not a significant risk factor of anemia during pregnancy (Table 3). In addition, malaria parasites detected during pregnancy and treatment with artemether-lumefantrine during pregnancy were not associated with a significant risk of having anemia (Table 3). In multivariate analysis, pregnant women who had at least primary education had significantly reduced 26% risk of having anemia compared to those without formal education (aHR, 0.74 [95% CI 0.58, 0.96]) (Figure 4, Table 3).

### 2.4. Adverse Birth Outcomes

Out of 430 participants followed until delivery, 114 (26.5%, 95% CI: 22.3, 30.7) had at least one adverse birth outcome, including composite of LBW, fetal anemia, still-birth, spontaneous abortion, and premature birth (Table 4). Fetal anemia and low birth weight were the most frequent adverse birth outcomes with the prevalence of 18.1% (78/430) and 10.9% (46/423), respectively (Table 4). Eight women (1.9%) delivered through cesarean section.

#### 2.4.1. Factors Associated with Any Adverse Birth Outcomes

Histopathological placental malaria was found to significantly double the odds of having any adverse birth outcome both in univariate (OR 1.99 [95% CI 1.01, 3.92], *p* = 0.047) and multivariate analysis (aOR 2.10 [95% CI 1.06, 4.16]), *p* = 0.034). Women who received more than or equal to three SP doses did not have a significantly reduced risk of having the composite outcome of any adverse birth outcome compared to those women who used sub-optimal (less than three) SP doses (OR 0.74 [95% CI 0.48, 1.15], *p* = 0.18). Having asymptomatic malaria during pregnancy and treatment with artemether-lumefantrine, being adolescent pregnant woman (age < 20 years), lack of education, and being primigravida were not associated with significantly higher odds of having any adverse birth outcomes (OR [95% CI]: 1.27 [0.75, 2.15] *p* = 0.38; 1.14 [0.67, 1.93] *p* = 0.63; 1.15 [0.67, 1.96] *p* = 0.61; and 1.19 [0.73, 1.93] *p* = 0.49) respectively.

#### 2.4.2. Factors Associated with LBW

The use of more than or equal to three SP doses was associated with a 66% reduced risk of LBW, both in univariate and multivariate analyses (Table 5). Neonates from women who received optimal SP doses (more than or equal to three SP) had higher mean birth weight (195 grams) compared to those who received sub-optimal (less than three SP) SP doses (95% CI 110, 279) *p* < 0.001. In addition, presence of histopathological placental malaria was significantly associated with LBW (aOR, 2.95; 95% CI; [1.20, 7.24] *p* = 0.018). Being primigravida, an adolescent (age < 20 years), and no education were not associated with significantly higher odds of LBW. Having malaria during pregnancy and treatment with artemether-lumefantrine was not associated with significant odds of LBW (Table 5).

### 2.5. Safety and Tolerability Outcomes

Nausea was the most common (5.4% [24/417]) reported adverse drug-related event. One participant reported moderate skin rash a few days after the first IPTp-SP dose, and IPTp-SP was stopped for this participant. Two participants vomited a few hours after the first dose of IPTp-SP. The participant whose IPTp-SP was interrupted due to skin rash was closely monitored throughout pregnancy and was screened for malaria at each visit. No maternal death or newborn congenital anomalies were observed at delivery and at 6 weeks post-delivery follow up.

## 3. Discussion

Malaria endemic countries in sub-Saharan Africa have adopted and integrated the WHO recommended monthly single-dose IPTp-SP as part of routine antenatal care to be given as direct-observed-therapy at each scheduled visit until delivery. Despite this effort, malaria in pregnancy remains a public health problem in the continent. Because of parasite resistance, SP is no longer used to treat malaria infection in many African countries, including Tanzania, but the drug is still used to prevent malaria during pregnancy. Considering the current widespread SP resistance, monitoring the effectiveness of IPTp-SP in preventing malaria during pregnancy is critical. In this prospective observational study, we assessed the effectiveness of IPTp-SP for the prevention of malaria in pregnancy and adverse birth outcomes from a moderate malaria transmission rural area in Southeast Tanzania. We report substantial rates of histopathological placental-malaria (9.4%), any parasitemia (placental, cord or maternal) at delivery (20.9%), and parasitemia during pregnancy after initiation of IPTp-SP (16%). Additionally, significant association of histopathological placental malaria with adverse birth outcomes, in particular, LBW was found. Furthermore, we found no significant effect of receiving more than or equal to three doses of IPTp-SP in preventing placental malaria, but it significantly improved birth weights. In general, our finding indicates that although its malaria protection is compromised, more than or equal to three doses of IPTp-SP is safe and effective in reducing adverse birth outcome, particularly LBW.

The overall incidence of symptomatic malaria and parasitemia during pregnancy after initiating IPTp-SP in this study was 2.8% and 16%, respectively. Primigravida were found to have increased susceptibility and the burden of malaria detected throughout pregnancy compared to multigravida. This may suggest the need for more strategies targeting this vulnerable group to improve their health and birth outcomes. The gravidity-dependent differences in susceptibility to malaria during pregnancy is thought to be related to the development of immunity specific to placental malaria in the second and subsequent pregnancies [23,24].

The overall prevalence of malaria in Tanzania has substantially declined from 14% in 2015 [25] to 7.3% in 2018 [26]. However, the prevalence of placental malaria (9.4%) found in this study corresponds to the 8% prevalence found in the same study area reported previously in 2014 [27]. This may indicate that the burden of malaria among pregnant women in the setting has remained stable despite the provision of IPTp-SP. The findings of this study could probably suggest limitations on malaria-preventive strategies, including IPTp-SP in the study area. While the need to continue strengthen the existing strategies is inevitable, new innovative strategies are urgently needed to optimize malaria prevention during pregnancy.

Delayed initiation of ANC could be the limiting factor for receiving optimal IPTp-SP (more than or equal to three) doses in Tanzania [28], as also revealed in this study. Usually, ANC serves as a means for providing necessary services during pregnancy, including the provision of insecticide-treated bed nets and IPTp-SP for malaria prevention. Late initiation of ANC may lead to the late provision of IPTp-SP and insecticide-treated bed nets. As a result of the late initiation of IPTp-SP, pregnant women might be exposed to malaria in pregnancy, which may consequently lead to placental infection and adverse birth outcomes [29].

In this study, we found no significant association of histopathological placental malaria with the number of IPTp-SP dose received (Table 3). This may indicate lower effectiveness of SP in clearing or preventing new malaria infection, which is probably due to parasites resistance to SP in the study area. Our finding is in line with a previous report from the neighboring Uganda, which concluded that the efficacy of SP to clear peripheral parasites and prevent new infections during pregnancy is compromised in areas with >90% prevalence of SP resistance marker Pfdhps540E [30], which is also the case in Tanzania (90.4%) [20,31]. Similar to our findings, the Ugandan study reported the beneficial impacts of IPTp-SP on birth weight. Although in 2015 the WHO Malaria Policy Advisory Committee suggested that IPTp-SP remains effective in areas where quintuple-mutant haplotypes of *P. falciparum* to SP are highly prevalent, it stresses the need for further research investigating the relationship of SP resistance markers and IPTp effectiveness [32]. The observed substantial rates of parasitemia during pregnancy or at delivery in this study provide further evidence for the limited effectiveness of IPTp-SP in preventing malaria during pregnancy in Tanzania. This highlights the need to search for an alternative strategy and/or safe antimalarial medicine with a longer half-life to substitute SP for IPTp and reach the most vulnerable women early in pregnancy to prevent malaria and improve pregnancy outcomes in sub-Saharan Africa.

In this study, placental malaria was found to significantly increase the risk of any adverse birth outcomes. There was also a trend towards increased risk of LBW with placental malaria, which reached statistical significance in the multivariate model. The effect of placental malaria on the adverse birth effects was hypothesized to be due to fetal growth restriction resulting from placental insufficiency, which occurs when infected red blood cells sequester in the intervillous space causing subsequent inflammation and pathological changes [33,34].

Our results indicate improved birth weight associated with the uptake of more than or equal to three IPTp-SP doses. Significantly, IPTp-SP 3+ was associated with improved birth weight and reduced the risk of LBW compared to lower doses of SP taken during pregnancy. Similar studies have reported dose-dependent IPTp-SP impact on birth weight [11,30,35]. The effect of malaria on LBW is well known and has been one of the reasons for IPTp-SP use in endemic areas. The improved birth weight might be due to reduced load of parasitemia and parasites in the placenta associated with IPTp-SP 3+. The antimicrobial activity of sulfadoxine could also prevent some potential bacterial infections during pregnancy and result in improved maternal and fetal health [36,37]. This might have also partially contributed to improved birth weight. Improving birth weight and reducing the risk of LBW has been one of the fundamental bases for advocating IPTp-SP 3+ policy [38]. Therefore, our findings add to a growing body of literature, indicating that IPTp-SP 3+ is still effective at reducing the risk of LBW and improves birth weights in the settings of moderate malaria transmission. Since LBW is associated with infant mortality, strategies to reduce LBW could help the achievement of Sustainable Development Goal number 3.2 (SDG 3.2), which advocates for reducing infant mortality by the end of 2030. Therefore, innovative strategies to improve IPTp-SP 3+ coverage in malaria-endemic regions should be searched and enforced.

In this study, a high prevalence of anemia (73.8%) during pregnancy was found. Despite the provision of mebendazole at every visit and daily iron/folic supplementation, the majority of participants who had anemia at enrolment were also found to be anemic at delivery. Several factors, including poor adherence to iron/folic supplements and poor nutrition, may be the reasons for high anemia among the participants. In addition, initiation of iron-folic supplementation late in the second trimester may not improve anemia during pregnancy [39]. Early initiation of iron/folic acid in the first trimester may help to control the public health burden of anemia among pregnant women.

IPTp-SP was generally safe and tolerated by pregnant women. Although we only included pregnant women from the second trimester, the incidences of nausea reported should be interpreted with caution because nausea is usually common during pregnancy, especially in the first trimester. In a study done in the northeast of Tanzania, the use IPTp-SP was associated with decreased cord blood hemoglobin and increased the risk of fetal anemia, which was hypothesized to be due to in utero SP exposure, thus suppressing fetal hematopoiesis [40]. However, in our study, we did not find an association between fetal anemia with a higher number of SP doses (more than or equal to three IPTp-SP), indicating that IPTp-SP is still safe.

The study has some limitations. This study was done in an area with moderate malaria transmission intensity; therefore, the findings might not be generalizable to areas with either higher or lower transmission intensities. In addition, the parasites infecting pregnant women at delivery were not genotyped to demonstrate that malaria occurring despite IPTp-SP is due to mutant-resistant strains of *P. falciparum*. However, the strength of this study was that the interventions were given as DOT, and thus, assured the precision with the number of SP doses taken in relation to the outcomes. The study also employed various malaria detection techniques, including mRDT, microscopy, placental histology, and real-time PCR. Therefore, the rates of malaria parasitemia reported in this study reflect the actual burden of malaria among pregnant women in the study area.

## 4. Materials and Methods

### 4.1. Ethical Statement

The study received ethical approval from the Institutional Review Board of the Tanzania National Institute for Medical Research (NIMR) (NIMR/HQ/R.8a/Vol.IX/2342) and the Muhimbili University of Health and Allied Sciences (MUHAS) (2016-06-07/AEC/Vol.XI/2). Permission to conduct the study was sought from the District Executive Director, District Medical Officer, and the Kibiti Health Center Medical Officer in charge before study commencement. Written informed consent was obtained from all participants.

### 4.2. Study Design and Setting

This was a prospective observational study conducted from January 2017 to May 2019 at Kibiti Health Center in Southeast Tanzania. Kibiti Health Center is located in Kibiti District, Coast Region. Kibiti is an area with moderate malaria transmission.

### 4.3. Study Participants

A prospective cohort of 500 pregnant women was monitored from first attendance to the ANC through delivery. The inclusion criteria were: pregnant women attending ANC for the first time, with a gestational age of at least 13 weeks, mRDT negative, HIV negative, and age of ≥16 years. The exclusion criteria were clinical malaria or asymptomatic malaria detected by mRDT or severe anemia at enrollment, and history of malaria and receiving any anti-malarial drug for the past 1 month. Gestational age was ascertained by the history of last menstrual period and physical examination, which is the standard of care in Tanzania.

### 4.4. Study Procedures

Socio-demographic, medical, and obstetrics information, including medication and insecticide-treated nets use, as well as a clinical assessment, were recorded at enrollment. Weight, height, and body temperature were measured. Malaria rapid diagnostic test (mRDT Care start, ACCESS BIO Somerset, NJ, USA) was performed for screening. Malaria parasite mRDT positive pregnant women were treated with artemether-lumefantrine according to the national guidelines [41] and were excluded from the study. Malaria RDT negative women were enrolled and received three tables of sulfadoxine–pyrimethamine (SP) under directly observed therapy (DOT). Each SP tablet contained 500 mg of sulfadoxine and 25 mg of pyrimethamine. Participants also received single-dose mebendazole 500 mg as DOT and prescribed daily doses of ferrous sulfate (200 mg) with folic acid (0.4 mg) for the prevention of anemia. Hemoglobin concentration was assessed by HemoCue Hb 201+ analyzer (HemoCue AB Angelholm, Sweden). Those who had severe anemia at study enrollment were excluded from the study and referred for further management. Participants with moderate to mild anemia at baseline were treated with ferrous sulfate 200 mg given three times daily with daily folic acid 0.4 mg according to the national guidelines [42]. All participants were screened for HIV at enrollment. Long-lasting insecticide-treated nets were provided to all pregnant women at enrollment as recommended by the national malaria control program. The use of an insecticide-treated bed net was assessed at each antenatal care visit.

### 4.5. Study Participants Follow Up

Routine visits were conducted every 4 weeks, including the collection of blood for determination of hemoglobin concentration, and screening of malaria by mRDT. Dried blood spots were also collected on filter papers at each visit for malaria detection using real-time PCR. At each routine visit, participants received IPTp-SP and mebendazole under DOT, and any adverse events during the first week after receiving IPTp was recorded. Participants were encouraged to come to the clinic anytime they were ill. Those who visited the clinic in unscheduled visits were first attended by study clinicians, and those who presented with a documented fever (tympanic temperature, ≥37.5 °C) or history of fever in the previous 24 h had blood collected for malaria testing using mRDT and a thick blood smear for microscopy. If the tests were positive for parasites, malaria was diagnosed and treated with artemether-lumefantrine according to the national treatment guidelines [41]. Participants were also encouraged to deliver at the study clinic. At delivery, a standardized assessment form was completed, including gestational age; weight; sex; congenital anomalies; and biologic specimens were collected, including placental tissue, placental blood, cord blood, and maternal venous blood. Maternal venous blood, placental blood, and cord blood were collected in EDTA tubes. After delivery, participants were followed for 6 weeks where any adverse events, including congenital anomalies (also assessed at delivery), were assessed. All data were double entered into electronic case report forms database, Census and Survey Processing system version 7 (CSPro 7) (US Census Bureau, USA) developed by the data management unit at National Institute for Medical Research (NIMR) in Mwanza.

### 4.6. Sample Collection at Delivery

At delivery, maternal venous blood, blood from the placental intervillous space, and cord blood were collected. The placental blood was collected following incision into the maternal surface of the placenta. The blood samples were screened for malaria using mRDTs, microscopy, and real-time PCR. Two pieces of placenta biopsy from the maternal side (2 cm^3^) were collected within 30 min after delivery to avoid autolysis. The biopsies were immediately fixed with 10% buffered formalin into a container and stored at room temperature.

### 4.7. Diagnosis of Anemia

Peripheral finger-prick blood was used to determine hemoglobin level by using HemoCue Hb 201+ analyzer (HemoCue AB Angelholm, Sweden). Anemia was defined when the maternal hemoglobin level was <11 g/dL. Mild, moderate, and severe anemia was defined when maternal Hb level was 10–10.9 g/dL, 7–9.9 g/dL and <7 g/dL, respectively [43]. Fetal anemia was defined when the cord blood Hb was <12.5 g/dL [44].

### 4.8. Diagnosis of Malaria by Rapid Diagnostic Test (RDT) and Microscopy

Detection of malaria parasites was done from peripheral maternal blood at enrollment, at each visit and from the placenta, cord, and maternal venous blood at delivery. Malaria Pf/PAN (HRP2/pLDH) Ag Combo rapid diagnostic tests (RDTs) (Care start, ACCESS BIO Somerset, NJ, U.S.A) were used. RDTs were performed according to the manufacturer’s instructions and examined after 20 min following the addition of four drops of the wash buffer. The test detects four *Plasmodium* species responsible for malaria in human (*P. falciparum, P. ovale, P. vivax, and P. malariae)*. RDTs target *P. falciparum* antigens histidine-rich protein 2 (HRP2) and *Plasmodium* lactate dehydrogenase (pLDH) for *P. falciparum, P. ovale, P. vivax,* and *P. malariae*.

Screening for malaria parasites by microscopy was done to confirm clinical malaria for febrile pregnant women during irregular visits as well as in all women at delivery. Thick blood smears stained with 2% Giemsa were prepared and read by experienced laboratory technicians. A thick blood smear was considered to be negative when the examination of 100 high-power fields did not reveal asexual parasites and/or gametocytes.

### 4.9. Detection of Malaria by Histology

Placental tissues were processed for histological evidence of placental malaria according to the procedures described previously [45]. Two histopathological slides for each sample were prepared and read in duplicate by two independent readers, and the results were recorded on a standardized case report form. Discrepant readings were taken to an independent third reader, and conclusive results were based on two readers. The laboratory scientists who had no other roles in the study were blinded on the number of SP doses a participant received at delivery to minimize bias. Evidence for positive placental malaria was concluded when malaria parasites were observed, indicating active infection or malaria pigments were observed, indicating past infection.

### 4.10. Detection of Malaria by Real-Time Polymerase Chain Reaction (PCR)

Dried blood spots on filter papers obtained at each monthly visit and at delivery were punched, and three circles of 3 mm in diameter were used for DNA extraction using QIAamp DNA blood micro kit (Qiagen GmbH, Hilden, Germany) following the manufacturer’s recommendations. Real-time PCR was used to qualitatively detect Plasmodium infection (*P. falciparum, P. vivax, P. ovale, and P. malariae*) in the 7500 Fast Real-Time PCR system (Applied Biosystems), following a previously described protocol [46]. The master mix for a single reaction included species-specific probes and forward primers for all four Plasmodium species used in combination with a conserved reverse primer. The *P. ovale-, P. malariae-, P. vivax-,* and *P. falciparum*-probes (Applied Biosystems) were each labeled with a distinct fluorophore, and Mustang Purple was used as the reference dye [47]. The reaction was performed in duplicate in a final volume of 15 μL per well containing 3 μL DNA, 7.5 μL of TaqMan multiplex master mix (Applied Biosystems), 0.3 μL (10 μmol/L) of each species-specific forward primers, 0.75 μL (10 μmol/L) of the reverse primer, 0.15 μL (10 μmol/L) of each species-specific probe, passive reference dye Mustang Purple, and DNA/RNA-free water. The samples were run using a cut-off of 45 cycles to define positive samples, starting with 95 °C for 20 s, followed by the thermal cycles of 95 °C for 1 s and of 60 °C for 20 s. Standards, negative and species-specific positive controls were included on each plate. The assay was optimized to detect all species simultaneously.

### 4.11. Study Outcomes

The primary outcome was the prevalence of histopathological placental malaria (parasites and pigments). Secondary outcomes included incidence of symptomatic malaria during pregnancy, prevalence of parasitemia during pregnancy and at delivery detected by rapid diagnostic test, microscopy and/or real-time PCR, prevalence of maternal anemia (Hb < 11 g/dL) during pregnancy, any adverse birth outcomes (stillbirth, low birth weight (<2500 g), fetal anemia (cord blood Hb < 12.5 g/dL), preterm delivery (<37 weeks), and spontaneous abortion (<28 weeks gestational age)).

### 4.12. Data Management and Statistical Analysis

Data from the CSPro database were exported to excel for cleaning and then to Statistical Package for Social Sciences (SPSS) version 26 for analysis. All enrolled pregnant women were included in the analysis of malaria and anemia during pregnancy. Participants with primary outcome data were included in the analysis of outcomes at delivery. Descriptive analysis used n (%) for binary and categorical variables, and mean (SD) or median (minimum and maximum) if the data were skewed for continuous variables. For continuous variables, normal distribution was assumed and checked by Shapiro–Wilk test, skewness, visual inspection of their histograms, normal Q-Q plots, and box plots. Independent *t*-test was used to compare mean birth weight between sub-optima and optimal IPTp-SP doses groups at delivery. Results were expressed as the mean difference with 95% CI. For categorical variables, results were analyzed as percentage prevalence with corresponding 95% confidence intervals and the *p*-values. Independent variables were compared by χ2 test or Fisher’s exact test. Proportions of anemia at enrollment and at delivery were compared by McNemar test. Malaria during pregnancy was defined as any malaria parasite detected while on IPTp-SP during pregnancy. Factors associated with the detection of malaria and anemia over time [time-to-becoming positive] were analyzed using Cox proportional hazards regression model, with the time of follow-up as the main covariate for evaluating the proportion of positive samples as the outcome. For those who developed malaria (event = 1), time-to-event was the respective last visit month on which malaria was detected (which corresponds to the number of SP doses received). Participants who did not develop malaria (event = 0) were censored, and the respective time-to-event was set as the last follow up visit month. Cohen’s kappa coefficient was used to test agreement of positive and negative parasitemia readings. Binary logistic regressions were used to identify factors associated with placental malaria, any adverse birth outcomes, LBW, and any malaria at delivery. Variables with *p* ≤ 0.2 in univariate analysis were included in multivariate model. The goodness of fit of the model was checked by Hosmer and Lemeshow test (*p* > 0.05). Crude and adjusted odds ratio with their respective *p* values were presented. The significance level was set at 0.05 and the confidence level at 95%. All *p* values were two-sided, and a *p*-value of less than 0.05 was considered to indicate statistical significance.

## 5. Conclusions

We report substantial rates of asymptomatic malaria (parasitemia) and placental malaria among pregnant women living in a moderate malaria transmission area of Tanzania despite receiving IPTp-SP. There was no significant association of IPTp-SP 3+ in preventing histopathological placental malaria in the study area. Placental malaria is significantly associated with increased adverse birth outcomes, particularly low infant birth weight. Nevertheless, IPTp-SP is safe, tolerated, and more than or equal to three SP doses improves birth weight compared to less than three SP doses in an area of moderate malaria transmission. The observed high rates of anemia during pregnancy and adverse birth outcomes highlight the need to strengthen malaria control and prevention strategies in the setting. Although IPTp-SP is still effective in reducing adverse birth outcomes, the observed parasitemia at delivery in one-fifth and histopathological placental malaria in one-tenth of pregnant women receiving IPTp-SP indicate the need to optimize malaria prevention strategy in pregnancy and search for alternative drugs for IPTp.

## Figures and Tables

**Table 1:** Participants’ sociodemographic characteristics at enrollment and during pregnancy.

Characteristic	n	%
**Age (years)**		
Mean age (SD)	26.5 (7.3)	-
16–19 (adolescent)	85	20.4
20–34 (young adult)	251	60.2
>35 (adult)	81	19.4
**Gestational age at enrollment (weeks)**		
Median gestational age (min-max)	22 (14–32)	-
Early attendance (13–20 weeks)	193	46.3
Late attendance (21–32 weeks)	224	53.7
**Trimesters at enrollment**		
Second trimester (13–27 weeks)	383	91.8
Third trimester (≥28 weeks)	34	8.2
**Gravidity at enrollment**		
Primigravida	107	25.7
Secundigravida	92	22
Multigravida	218	52.3
Parity (number of live children)		
Median (range)	2 (0–9)	-
**Education level**		
Primary complete & above	336	80.6
No formal education	81	19.4
Insecticide treated bed net (ITN) use	305	73.1
**Hemoglobin** (g/dl), mean (SD)	10.3 (1.4)	-
**Body** temperature, (℃) median (range)	37 (34–37)	-
**Body weight**, kg, median (min-max)	54 (39–95)	-
Height, cm, median (min-max)	151 (142–164)	-
**Number of SP doses received**		
Median SP doses (min-max)	3 (1–5)	-
Received optimal SP doses (≥3 doses)	256	61.4
Received sub-optimal SP doses (<3 doses)	161	38.6
**Insecticide-treated bed net** (ITN) use during pregnancy	405	97.1

**Table 2:** Malaria outcomes at delivery and during pregnancy.

	Method	n	Proportion in % (95% of CI)
**Placental tissue**	Histology (parasites or pigments)	39/417	9.4 (6.9, 12.6)
Placental **blood**	RDT	11/417	2.6 (1.4, 4.7)
	microscopy	10/417	2.4 (1.3, 4.4)
	PCR	38/417	9.1 (6.5, 12.3)
	RDT, microscopy or PCR	38/417	9.1 (6.5, 12.3)
Cord blood	RDT	11/417	2.6 (1.4, 4.7)
	microscopy	10/417	2.4 (1.3, 4.4)
	PCR	21/417	5 (3.3, 7.6)
	RDT, microscopy or PCR	22/417	5.3 (3.3, 7.9)
**Maternal blood at delivery**	RDT	12/417	2.9 (1.6, 5.0)
	microscopy	9/417	2.2 (1.1, 4.1)
	PCR	41/417	9.8 (7.3, 13.1)
	RDT microscopy or PCR	42/417	10.1 (7.4, 13.4)
**Infection during pregnancy**	Symptomatic malaria	14/500	2.8 (1.5, 4.7)
	Positive RDT at ANC visits	40/500	8 (5.8, 10.7)
	Positive RDT and/or PCR at ANC visits	80/500	16 (12.9, 19.5)

**Table 3:** Factors associated with histopathological placental malaria (using logistic regression analysis), malaria parasites detected during pregnancy and anemia during pregnancy while on IPTp-SP using cox regression analysis (n = 417).

Dependent Variables	*Characteristics*	Univariate Analysis			Multivariate Analysis	
		N (%)	OR (95% CI)	*p* Value	aOR (95% CI)	*p* Value
Histopathological placental malaria	**Age (years)**					
	16–20	12 (14.1)	1		1	
	20–34	14 (5.6)	0.36 (0.16, 0.81)	0.014	0.36 (0.16, 0.83)	0.016
	≥35	13 (16)	1.16 (0.50, 2.73)	0.73	1.11 (0.47,2.62)	0.82
	**Gravidity**					
	Primigravida	12 (11.2)	1.32 (0.65, 2.72)	0.44		
	Multigravida	27 (8.7)	1			
	**Education level**					
	No education	9 (11.1)	1	0.28		
	Primary and above	30 (8.9)	0.58 (0.22, 1.54)			
	**Malaria parasites detected during pregnancy**					
	Yes	11 (13.8)	1.76 (0.84, 3.71)	0.14	1.64 (0.77, 3.51)	0.2
	No	28 (8.3)	1		1	
	**Number** of SP doses			0.1		0.14
	<3 SP doses	10 (6.2)	0.52 (0.25, 1.10)		0.56 (0.26, 1.20)	
	≥3 SP doses	29 (11.3)	1		1	
			**HR (95% CI)**		**aHR (95% CI)**	
Malaria parasites detected during pregnancy	**Age (in years)**					
	16–20	18 (21.2)	1			
	20–34	45 (17.9)	0.85 (0.49, 1.46)	0.55		
	≥35	*17 (21*)	0.94 (0.49, 1.83)	0.86		
	**Gravidity**					
	Primigravida	28 (26.2)	1.66 (1.05, 2.63)	0.03		
	Multigravida	52 (16.8)	1			
	**Education level**					
	No education	17 (21)	1	0.91		
	Primary and above	63 (18.8)	0.97 (0.55, 1.70)			
Anemia during pregnancy	**Age (in years)**					
	16–20	79 (77.5)	1		1	
	20–34	223 (74.1)	0.87 (0.67, 1.13)	0.3	0.94 (0.69, 1.28)	0.71
	≥35	67 (69.1)	0.73 (0.52, 1.01)	0.06	0.76 (0.51, 1.13)	0.18
	**Gravidity**					
	Primigravida	101 (78.9)	1	0.09	1	0.29
	Multigravida	268 (72)	0.82 (0.65, 1.03)		0.86 (0.64, 1.14)	
	**Education level**					
	No education	78 (80.4)	1	0.08	1	0.025
	Primary and above	291 (72.2)	0.80 (0.62, 1.03)		0.74 (0.58, 0.96)	
	**Malaria parasites detected during pregnancy**					
	Yes	73 (91.3)	1.16 (0.54, 2.53)			
	No	296 (87.8)	1	0.7		

HR—crude Hazard Ratio (time to event from inclusion and first IPTp-SP dose); aHR—adjusted Hazard *Ratio (age, gravidity, and level of education); CI—Confidence Interval; OR—crude odds ratio; aOR—adjusted odds ratio (age, number of SP doses and malaria parasite detected during pregnancy).

**Table 4:** Overall adverse birth outcomes.

Outcome Variables	n/N	% (95% CI)
Any adverse birth outcomes	114/430	26.5 (22.3, 30.7)
LBW (birth weight <2500 g)	46/423	10.9 (8.3, 14.2)
Premature birth (<37 weeks)	10/430	2.3 (1.3, 4.2)
Still birth	6/430	1.4 (0.6, 3.0)
Spontaneous Abortion (<28 weeks)	1/430	0.2 (<0.01, 1.3)
Fetal anemia (<12.5 g/dL)	78/430	18.1 (14.8, 22.1)

**Table 5:** Factors associated with LBW.

Characteristics	LBW n (%)	Univariate Analysis		Multivariate Analysis	
		OR (95% CI)	*p* Value	aOR (95% CI)	*p* Value
**Age (years)**					
<20	13 (15.3)	1		1	
20–34	28 (11.2)	0.70 (0.34, 1.41)	0.32	0.87 (0.37, 2.06)	0.76
≥35	5 (6.20)	0.36 (0.12, 1.07)	0.07	0.45 (0.13, 1.58)	0.21
**Gravidity**					
Primigravida	16 (15)	1.64 (0.86,3.15)	0.14	1.30 (0.58, 2.93)	0.52
Multigravida	30 (9.7)	1		1	
**Level of education**		1			
No education	12 (14.8)	0.55 (0.76,3.14)			
Primary and above	33 (9.9)	1	0.23		
**Malaria parasites detected during pregnancy**					
Yes	11 (14.9)	1.38 (0.67, 2.84)			
No	35 (10.2)	1	0.39		
**Malaria parasites detected during pregnancy**					
**<3 SP doses**	5 (19.2)	1.91 (0.52, 6.94)	0.33		
**≥3 SP doses**	6 (11.1)	1			
**Placental malaria**					
Yes	8 (20.5)	2.31 (0.99, 5.38)	0.05	2.95 (1.20, 7.24)	
No	38 (10.1)	1		1	0.018
**Number of SP doses**			0.009		
<3 doses	26 (16.3)	1		1	
≥3 doses	20 (7.8)	0.44 (0.24, 0.82)		0.41 (0.22, 0.78)	0.007

OR—crude odds ratio; aOR—adjusted odds ratio (age, gravidity, histopathological placental malaria and number of SP doses received); LBW—Low birth weight; CI—Confidence Interval.
